# Innovative Medial Cushioning Orthoses Affect Peroneus Longus Electromyographic Activity during Running

**DOI:** 10.3390/jcm11051339

**Published:** 2022-02-28

**Authors:** Ruben Sanchez-Gomez, Alvaro Gomez-Carrion, Carlos Martinez-Sebastian, Luis Alou, David Sevillano, Almudena Nuñez-Fernandez, Paola Sanz-Wozniak, Blanca de la Cruz-Torres

**Affiliations:** 1 Nursing Department, Faculty of Nursing, Physiotherapy and Podiatry, Universidad Complutense de Madrid, 28040 Madrid, Spain; alvaroalcore@hotmail.com (A.G.-C.); carlos_mar_seb@hotmail.com (C.M.-S.); almnun01@ucm.es (A.N.-F.); paolsanz@ucm.es (P.S.-W.); 2 Microbiology Division, Department of Medicine, Universidad Complutense de Madrid, 28040 Madrid, Spain; luisalou@ucm.es (L.A.); dsevill@med.ucm.es (D.S.); 3 Department of Physiotherapy, University of Seville, 41009 Seville, Spain; bcruz@us.es

**Keywords:** orthoses, peroneus longus, supination, surface electromyography

## Abstract

Background: Over-supination processes of the foot and ankle involving peroneus longus (PL) damage during running sports have been treated conservatively with passive control tools, such as tapes, braces, or external ankle supports, but the effect of orthoses with typical lateral wedging orthoses (TLWO) on the muscular activity of PL during running remains unclear. Here we investigate the effects of innovative medial cushioning orthoses (IMCO) on PL activity during the full running gait cycle. In addition, we wished to ascertain the effects of innovative medial cushioning orthoses (IMCO) on PL activity during running. Methods: Thirty-one healthy recreational runners (mean age 34.5 ± 3.33) with neutral foot posture index scores, were selected to participate in the present study. They ran on a treadmill at 9 km/h wearing seven different orthoses (NRS, IMCO 3 mm, IMCO 6 mm, IMCO 9 mm, TLWO 3 mm, TLWO 6 mm and TLWO 9 mm), randomly performed on the same day while electromyographic activity of the PL muscle was recorded. Statistical intraclass correlation coefficient (ICC) to test reliability was carried out and the Wilcoxon test with Bonferroni’s correction was developed to analyze the differences between the conditions. Results: the reliability of all assessments showed data higher than 0.81, that is, “almost perfect reliability”; all EMG PL values wearing either TLWO or IMCO showed a statistically significant reduction versus NRS during the fully analyzed running gait cycle; the highest difference was set on NRS 23.08 ± 6.67 to TLWO 9 mm 17.77 ± 4.794 (*p* < 0.001). Conclusions: Muscular EMG activity of the PL during the full running gait cycle decreases when wearing either TLWO or IMCO relative to NRS; therefore, these orthoses could be prescribed to treat the strain and overload pathologies of PL. In addition, IMCO—as it less thick, compared with TLWO—can be used when aiming to achieve better running economy.

## 1. Introduction

Lateral ankle sprains and peroneal tendon disorders are common pathologies among recreational runners [1]. The lateral ligament complex and peroneus apparatus contribute to countering the normal supination moments in the first phase of the human gait [2], named the heel contact phase. There are multiple conservative interventions to treat lateral ligament ankle disorders, depending on the ankle sprain level and phase of its evolution [3]: compression, ice, non-steroidal anti-inflammatory medications, elevation and rest are prescribed to avoid swelling and to reduce the pain in the first inflammation phase; during the second proliferation phase, motion rehabilitation to recover the normal range of motion; and in the third phase, neuromodulation and strengthening to reach the normal level of sport competition or daily activity [4]. Furthermore, external ankle supports (such as an ankle brace or tape) for up to 1 year after an ankle sprain have been used to assist functional rehabilitation in runners [5,6]. However, plantar foot orthoses have not been considered as part of the efforts to quickly improve these ligament issues.

In addition to ankle ligament injuries, peroneal longus (PL) tendon damage is known to occur when a lateral ankle sprain is present and in over-supinated feet or hindfeet varus [7]. Those with chronic lateral ankle instability have higher PL electromyographic (EMG) activity than neutrals [8]. The tendon falls into three broad forms: tendinitis/tendinosis plus tenosynovitis, tendon subluxation/dislocation, and tendon splits/tears [9]. Anti-inflammatory drugs, rest, or activity modification are typically prescribed; elsewhere orthoses with typical lateral wedging orthoses (TLWO) or lateral forefoot posting have been proposed to solve slight or mild cases [10,11], tilting the hindfoot to the valgus position to diminish the strain of the lateral tissues of the ankle. However, while the effects of typical supination orthoses on peroneus longus or tibial anterior muscular behaviour have been assessed in some EMG studies [12,13], the EMG of lateral pronation orthoses on PL activity remains unclear, especially during running; in addition, no studies have investigated the effects of innovative medial cushioned orthoses (IMCO) without any lateral enhancement material combined with a medial soft stuffed material on the EMG activity cost of PL muscle during running activity. 

Therefore, the purpose of the present study was to investigate the EMG changes of PL activity during running wearing two kinds of orthoses: TLWO of 3, 6, and 9 mm versus IMCO of 3, 6, and 9 mm, relative to neutral running shoes (NRS) without orthoses. We hypothesized that IMCO could decrease the muscular activity of PL more than TLWO or NRS during the full running gait cycle.

## 2. Materials and Methods

The present study was reviewed and approved by the review board at Nuestra Señora de Valme Hospital (certificate number 2115 N20). Ethical and Human guides have been followed according to the Declaration of Helsinki; all participants signed informed consent before starting the study. 

### 2.1. Study Design and Sample Size

The appropriate sample size needed for the present study was calculated by the statistics unit of Universidad Complutense de Madrid, Spain. The aim was to compare the EMG changes of PL muscle activity between TLWO of 3, 6, and 9 mm versus IMCO of 3, 6, and 9 mm relative to NRS during running. In previous studies of gastrocnemius lateralis EMG activity during running, a value of 25.96 ± 4.68 millivolts (mV) wearing novel orthoses has been reported (compared to 22.27 ± 2.51 mV wearing classic shoes) (*p* < 0.05). Considering a statistical power of 80%, a 95% confidence interval (CI), β = 20%, and α = 0.05, it was estimated that 31 subjects would be needed in this study. The Observational Studies in Epidemiology (STROBE) [3] criteria and randomly consecutive sampling techniques were followed throughout this study.

### 2.2. Participants

The following inclusion criteria were followed to select the participants: (1) healthy women and men people ranging between 18 and 30 years old; (2) recreational rearfoot strike pattern-runners that perform 3 days per week training sessions with at least 1 year of experience; and (3) neutral foot posture index (FPI) [14]. The exclusion criteria were (1) to have any pain or any lower limb injury at the time of the test or 1 year ago; (2) to be in treatment with any medication; and (3) to have any restrictive joint mobility on feet or lower limb far from valid values [15,16]. Body max index (BMI) = weight (kg)/height (m^2^) was calculated, avoiding hypothetical bias on data recollection or interpreting.

### 2.3. Instruments and Assessments

A NeuroTrac Simplex Plus (Verity Medical Ltd., Braishfield, UK) EMG electronic device with a USB-Bluetooth was used to study the superficial EMG contraction [17,18] of PL muscle fibres during running trials; 0.2 mV to 2000 mV was the range of record of the device, with a sensitivity of 0.1 mV root mean square (RMS), 10 m of free wireless (via Bluetooth) connection range, and an accuracy of 4% of the reading from mV ± 0.3 mV to 200 Hz with a bandpass filter of 18 Hz ± 4 Hz to 370 Hz ± 10% for readings below 235 mV. 

The signal was detected with self-adhesive circular surface electrodes made on high-quality hydrogel with conductive carbon film and 30 mm dia-round size; the information was sent under a unidirectional radioelectric secure connection to the receiver module and filtered automatically by the NeuroTrac software (Verity Medical Ltd., Braishfield, UK). After that, the computer digitally transformed it by the software to generate the activity pattern values of each electrode. 

### 2.4. Materials

**Figure 1:**
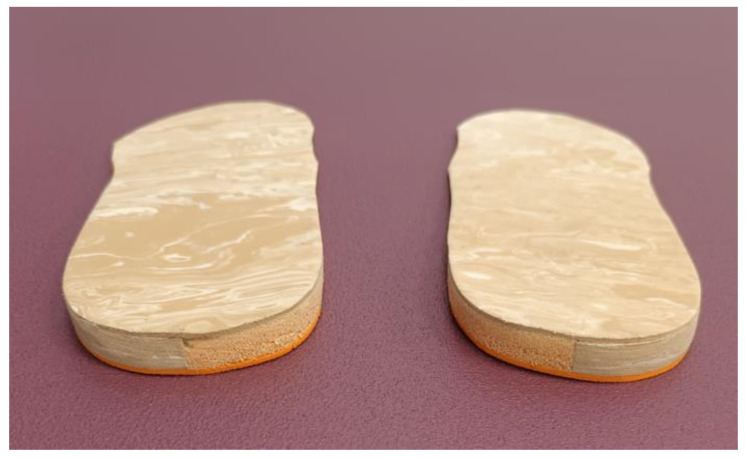
Innovative medial cushioned orthoses (IMCO) with medial cushioning casting filled with Poron (Microban).

**Figure 2:**
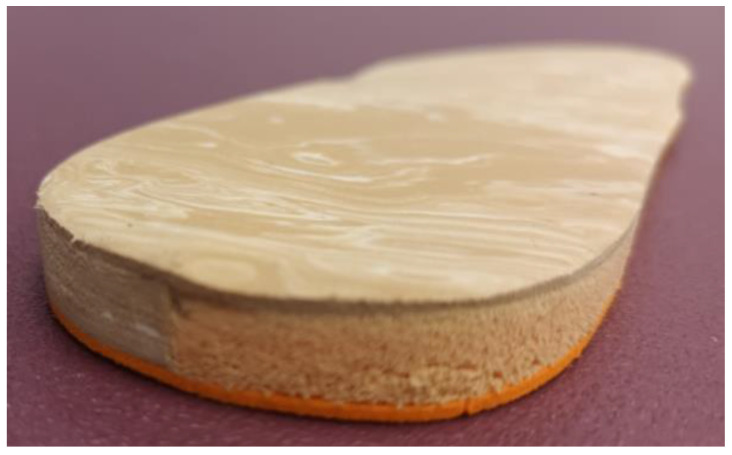
Detail of medial cushioning casting filled with Poron (orange material) set on rear-medial side of a left innovative medial cushioned orthotic (IMCO).

IMCO was made with a flat sheet of 3 mm density and high hardness of ethylene-vinyl acetate (EVA) and with a medial cushioning casting placed from the bisectrix of the rear part of the orthotic to its medial edge, filled with viscoelastic rubber of Poron (Microban) [19]. Covering the superficial layer of the orthotic, 1 mm thickness of low hardness EVA was used (Figure 1 and Figure 2). A flat sheet of 6 and 9 mm with the same design and materials were made to complete the IMCO.

**Figure 3:**
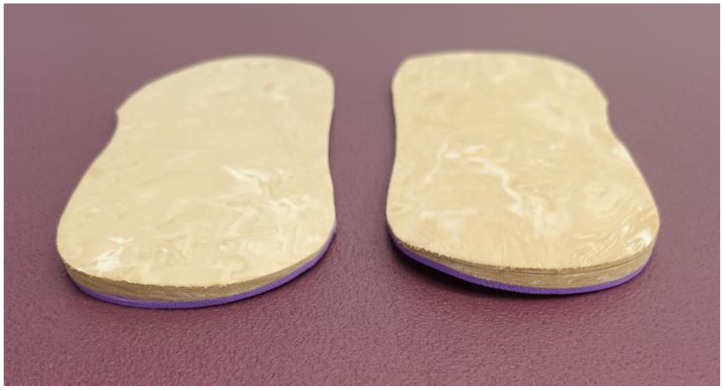
Typical lateral wedging orthoses (TLWO) with lateral enhancement of EVA on the rear side.

**Figure 4:**
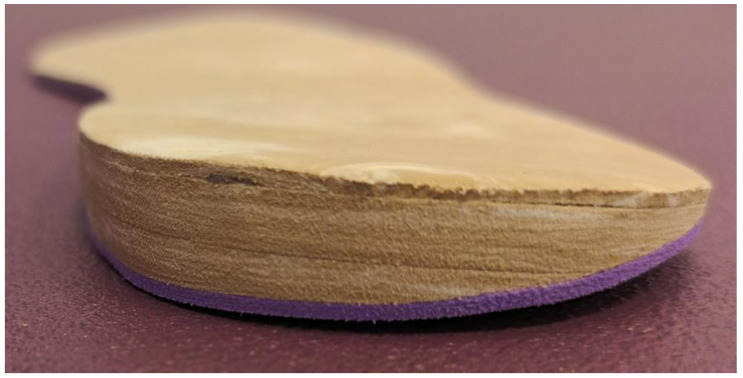
Detail of the enhancement of EVA set on rear-lateral side of a left typical lateral wedging orthotic (TLWO).

The TLWO was made with a flat sheet of 1 mm of ethylene-vinyl acetate (EVA) thickness of high hardness with a posting wedge of 3 mm EVA thickness, placed on the lateral rear of the orthotic (Figure 3 and Figure 4). Other 6 and 9 mm lateral wedge postings were made to complete the TLWO.

To avoid any influence on the normal foot biomechanics behavior, no more orthotic elements were added in the inserts; both left and right IMCO and TLWO orthoses were manufactured. The laboratory producer of these orthoses was an external company and was blinded during all stages of the study. The NRS used were “New Feel PW 100 M medium grey” (ref. number: 2018022). 

### 2.5. Procedure

An experienced podiatric clinician and researcher (Rubén Sánchez-Gómez) took the measurements of the participants. To set the proper location of the sensors on PL muscular belly, the researcher had to request each participant to do foot eversion against clinician resistance for a few seconds; then, self-sticking surface electrodes were placed on the skin area where the most prominent bulge of the PL muscle was located, according to European recommendations for surface EMG [20]. Next, maximal eversion movement against the clinician’s hand resistance was done again for 5 s to set the maximal voluntary contraction needed to calibrate the device and normalise each trial’s EMG data amplitudes. 

#### Running Test

An automatic motorized treadmill (Domyos T520) was used to perform the running test. Subjects were requested to run once for 3 min at 5.7 km/h on the treadmill wearing NRS to become acclimatized [21] and reduce the hypothetical bias of “new use-materials”. Then, the running test was performed at 9 km/h [22] under seven conditions (NRS, IMCO 3 mm, IMCO 6 mm, IMCO 9 mm, TLWO 3 mm, TLWO 6 mm, and TLWO 9 mm) randomly performed on the same day. The mean of the EMG PL muscle activity of the right leg was recorded for 30 s, three times (a total of 21 trials per participant), leaving 5 min for rest between each test [23]. To avoid a possible imbalance of the musculoskeletal system, the same orthotic conditions for each trial were placed on the left foot.

### 2.6. Statistical Analysis

Demographic variables were shown as means and standard deviations. The within-day trial-to-trial intraclass correlation coefficient (ICC) and standard error of measurement (SEM) were calculated [24] for all subjects under seven conditions to assess the reliability of the present research. According to Landis and Koch [25] estimations, the ICCs would be slight if it showed < 0.20; fair if 0.20–0.40; moderate if 0.41–0.60; substantial if 0.61–0.80; and almost perfect agreement if 0.81–1.00. In the present research, we considered ICCs of ≥0.81 to get the optimal scientific validity to support the results. The SEM was calculated to find the minimum detectable change (MDC) for all measurements. The normality of the sample was calculated using the Shapiro–Wilks test, considering normal distribution if *p* > 0.05.

To check the presence of the difference between conditions, a non-parametric Friedman test was used. To analyse these differences, the Wilcoxon test with Bonferroni correction was performed. Statistically significant differences were indicated when *p* < 0.05, with 95% CI.

## 3. Results

All present study participants were recruited from a podiatry clinic in Madrid (Spain) over 90 days (November 2020 to January 2021). Fifty-six subjects were asked to participate in the experiment and assessed for eligibility; twenty participants did not meet the requirements of inclusion criteria, and five were lost participants; ultimately, 31 participants (14 males and 17 females) were enrolled in the study; sociodemographic data are shown in Table 1.

The Shapiro–Wilks test showed a no-normal distribution of the sample (*p* < 0.05) and Friedman test showed that the values were different between the conditions (*p* < 0.05).

The reliability of the EMG muscle data during the full running activity cycle for the seven different conditions is shown as the ICC and SEM in Table 2. All recollected data were higher than the established 0.81, which suggest that values had “almost perfect reliability” [25]. The TLWO 9 mm reached the highest value with 0.993, and the IMCO 3 mm the lowest with 0.990. Regarding SEM, the IMCO 9 mm achieved the lowest value (0.372) and the TLWO 3 mm the highest (0.463). The MDC data showed the lowest value for the IMCO 9 mm (1.03) and the TLWO 3 mm was the highest (1.284). All MDC values were lower than the mean differences found between NRS, TLWO, and IMCO, and therefore, the data reach the optimal quality to be considered.

PL muscle EMG mean activities while running with NRS were compared to TLWO 3 mm, 6 mm, 9 mm and with IMCO 3 mm, 6 mm, and 9 mm (Table 3, with their *p*-values in Table 4).

All EMG values of TLWO and IMCO showed statistically significant reductions in respect to NRS; in detail, from NRS 23.08 ± 6.67 to TLWO 3 mm 18.23 ± 4.16 (*p <* 0.001), to TLWO 6 mm 17.9 ± 3.78 (*p <* 0.001) and to TLWO 9 mm 17.77 ± 4.794 (*p <* 0.001); from NRS 23.08 ± 6.67 to IMCO 3 mm 18.64 ± 4.40 (*p <* 0.001), to IMCO 6 mm 18.26 ± 4.26 (*p <* 0.001) and to IMCO 9 mm 18.13. ± 3.86 (*p <* 0.001). Concerning the comparisons between pair-to-pair, the TLWO/IMCO data also showed some statistically significant values, specifically for IMCO 3 mm (18.64 ± 4.40) versus IMCO 6 mm (18.26 ± 4.26) (*p* < 0.05) and IMCO 3 mm (18.64 ± 4.40) versus IMCO 9 mm (18.13 ± 3.86) (*p* < 0.05).

## 4. Discussion

In the present study, we aimed to determine the EMG activity reaction of the PL wearing NRS compared with IMCO and TLWO.

The use of lateral load deviations to treat peroneal disorders associated with rearfoot varus deformities are justified in multiple studies; in some cases, authors use lateral wedges to increase the lateral moments of the ankle [11,26,27] and/or subtalar joint [28] to alleviate medial knee osteoarthritis syndromes; in other cases, surgery procedures [29,30] are indicated to reduce varus hindfoot deformities to improve the strain of the PL and/or brevis and even to support the hyperlaxity of the rearfoot complex. Closer to our study, orthoses devices together with taping have been proposed [4] as an intervention to prevent ankle sprains; postural sway of acute ankle-supinated injuries has shown improvement wearing orthoses and they offered protection against reinjury, enhancing the ankle mechanoreceptors [31,32]; however, the effects that support the control inversion perturbations of ankle prevention and/or during/post-injury were not clear [5].

According to our results, the decreases in the EMG of the PL signal when wearing either TLWO (*p* < 0.001) or IMCO (*p* < 0.001)—regardless of their thickness—verify that these orthoses with pronator elements could be a conservative option to relax over-supination injuries that affect the tired or strained PL [8]. This follows the subtalar axis rotation equilibrium theory that argued the use of external forces on the lateral side of that axis to increase the pronated moments on the rearfoot [33] and similarly in line with the results of Moisan et al. [34,35], who assessed the PL muscle activity on subjects with cavus foot during walking with orthoses with lateral bar similar to our TLWO and obtaining similar discharged effects on PL muscle EMG amplitudes. 

Based on available research, we can say that there is a lack of references to compare the decrease of PL EMG activity when wearing some orthotic device during running; Konradsen et al. [2] concluded that an external ankle support could decrease the EMG of PL during running. Another study referred that the EMG of PL in pronated or valgus feet were decreased during walking [36] or running [37], postulating the general concept of pronation and the decrease of PL muscular activity. The study of Baur et al. [10] compared PL activity with and without wearing detorsion wedge forefoot on semirigid orthoses of polyurethane during running, in patients with ankle instability; the authors did not find any differences between groups, only a higher pre-activation of PL EMG activity versus the group of no-orthoses was recorded; these results contrast with ours, but these researchers used a forefoot element whereas the key of the peroneal control is in the rearfoot; moreover, they studied the EMG of the PL wearing full-shape-conformed orthoses, including the longitudinal medial arch, which could itself influence the muscular activity of the PL due to its passing through the plantar foot, whereas present research made flat-shaped non-conformed orthoses with only a rearfoot effect, to avoid this plausible slant.

Regarding comparisons between IMCO vs. TLWO, we found that the alleviating effects on muscular PL activity were essentially the same compared with NRS with the advantage that IMCO density and size were less than TLWO, which supposes a wishing condition to running economy, according to Ray et al. [38]. The soft material embedded in the medial side of the IMCO decreased PL activity, as occurred when wearing similar soft material on cushioning running shoes in other references [39,40] during a running-EMG test.

### Limitations

The assessed muscular activity of the PL results must be interpreted with caution because the EMG device used in the present study has high sensitivity, and the maximal voluntary isometric contraction test used to calibrate the signal device can vary between participants. 

Due to the deep location of the peroneus brevis muscular belly and the difficulty in reaching its muscular activity with the superficial EMG device used in the present study, the authors decided to rule out the assessment of this muscle.

The present results are not specifics to any phase of running gait, but to a mean value of a whole running gait cycle.

## 5. Conclusions

Foot orthoses with rearfoot lateral corrections to control the supination of the hindfoot have been a classical tool to treat ankle instability, but there has been a lack of studies addressing PL muscular activity wearing these orthoses during running. The present research has shown that wearing either IMCO or TLWO versus NRS decreases the EMG activity of the PL during a full running gait cycle in healthy subjects, with the advantage that IMCO offers less thickness and volume to users and hence a better running economy. Therefore, the prescription of foot orthoses with pronator elements on the rearfoot can be considered to treat pathologies involving PL overloads. To our knowledge, this is the first study to show the EMG effects of TLWO on PL activity, thereby enhancing the knowledge about the use of these kinds of orthopaedic corrections of the rearfoot, away from the unique balance of the rearfoot varus position.

## Figures and Tables

**Table 1:** Subject demographics.

Variable	*n* = 31 mean ± SD (95% CI)
**Age** (years)	34.5 ± 3.33 (33.32–35.67)
**FPI** (scores)	3.71 ± 0.19 (2.01–3.01)
**Weight** (kg)	62.6 ± 8.68 (59.54–65.65)
**Height** (cm)	162.25 ± 3.21 (161.11–163.38)
**BMI** (kg/m^2^)	21.38 ± 3.02 (20.31–22.44)

Abbreviations: SD = standard deviation; CI = confidence interval; FPI = foot posture index; BMI = body mass index.

**Table 2:** Reliability ICC of variables with “Neutral Running Shoes” versus “Typical Lateral Wedging Orthoses” (TLWO) and “Innovative Medial Cushioned Orthoses” (IMCO) of 3, 6 and 9 mm.

Variable	NRS			TLWO 3 mm			TLWO 6 mm			TLWO 9 mm		
	ICC (95% CI)	SEM	MDC	ICC (95% CI)	SEM	MDC	ICC (95% CI)	SEM	MDC	ICC (95%CI)	SEM	MDC
**Peroneus Longus** (mV)	0.995 (0.992–0.998)	0.455	1.262	0.988 (0.978–0.994)	0.463	1.284	0.988 (0.978–0.994)	0.419	1.162	0.993 (0.987–0.996)	0.4	1.126
**Peroneus Longus** (mV)	0.995 (0.992–0.998)	0.455	1.262	0.990 (0.981–0.995)	0.451	1.251	0.992 (0.985–0.996)	0.382	1.06	0.991 (0.984–0.995)	0.372	1.03

Abbreviations: ICC = intraclass correlation coefficient; CI = confidence interval; SEM = standard error of measurement; MDC = minimal detectable change; (mV) = millivolts; SO = shoe only; mm = millimeters.

**Table 3:** EMG signal amplitudes of the mean peroneus longus muscle activities between different study situations.

	**NRS**	**TLWO 3 mm**	**TLWO 6 mm**	**TLWO 9 mm**
**Variable**	mean (mV)	Mean (mV)	mean (mV)	mean (mV)
	± SD (95% CI)	± SD (95% CI)	± SD (95% CI)	± SD (95% CI)
	23.08 ± 6.67	18.23 ± 4.16	17.9 ± 3.78	17.77 ± 4.794
**Peroneus Longus**	(20.63–25.53)	(16.7–19.76)	(16.59–19.37)	(16.01–19.52)
	**NRS**	**IMCO 3 mm**	**IMCO 6 mm**	**IMCO 9 mm**
**Variable**	mean (mV)	Mean (mV)	mean (mV)	mean (mV)
	± SD (95% CI)	± SD (95% CI)	± SD (95% CI)	± SD (95% CI)
	23.08 ± 6.67	18.64 ± 4.40	18.26 ± 4.26	18.13 ± 3.86
**Peroneus Longus**	(20.63–25.53)	(17.027–20.25)	(16.7–19.83)	(16.71–19.55)

Abbreviations: mV = millivolts; NRS = neutral running shoes; TLWO = typical lateral wedging orthoses; IMCO = innovative medial cushioned orthoses; mm = millimeters; ±SD = standard deviation

**Table 4:** *p*-Values comparison of the mean peroneus longus muscle activities between different study situations.

***p*-Value NRS**	***p*-Value NRS**	***p*-Value NRS**	***p*-Value TLWO 3 mm**	***p*-Value TLWO 3 mm**	***p*-Value** **TLWO 6 mm**	***p*-Value TLWO 3 mm** **vs.** **IMCO 3 mm**	***p*-Value TLWO 6 mm** **vs.** **IMCO 6 mm**	***p*-Value TLWO 9 mm** **vs.** **IMCO 9 mm**
**vs.**	**vs.**	**vs.**	**vs.**	**vs.**	**vs.**			
**TLWO 3 mm**	**TLWO 6 mm**	**TLWO 9 mm**	**TLWO 6 mm**	**TLWO 9 mm**	**TLWO 9 mm**			
<0.001 **	<0.001 **	<0.001 **	0.518	0.189	0.531			
***p*-Value NRS**	***p*-Value NRS**	***p*-Value NRS**	***p*-Value IMCO 3 mm**	***p*-Value IMCO 3 mm**	***p*-Value** **IMCO 6 mm**			
**vs.**	**vs.**	**vs.**	**vs.**	**vs.**	**vs.**			
**IMCO 3 mm**	**IMCO 6 mm**	**IMCO 9 mm**	**IMCO 6 mm**	**IMCO 9 mm**	**IMCO** **9 mm**			
<0.001 **	<0.001 **	<0.001 **	<0.05 *	<0.05 *	0.666	0.142	0.383	0.131

Abbreviations: mV = millivolts; NRS = neutral running shoes; TLWO = typical lateral wedging orthoses; IMCO = innovative medial cushioned orthoses; mm = millimeters; ±SD = standard deviation; *p* < 0.05 * (95% CI) was considered statistically significant; *p* < 0.001 ** (95% CI) was considered statistically significant.

## Data Availability

All data supporting reported results can be request at R.S.-G. and the data will be provided.
